# Clinical symptoms and molecular investigations in 13 patients with Schnitzler syndrome identified at the single UK centre

**DOI:** 10.1186/1546-0096-13-S1-P38

**Published:** 2015-09-28

**Authors:** D Rowczenio, H Trojer, A Baginska, J Gillmore, A Wechalekar, P Hawkins, H Lachmann

**Affiliations:** 1 National Amyloidosis Centre, University College London, London, UK; 2 National Amyloidosis Centre, UCL Medical School, London, UK

## Introduction

Schnitzler syndrome (SchS) was first described in 1972 and to date 281 cases have been reported. SchS is an adult onset, apparently acquired disease, which clinically closely resembles CAPS including the response to IL-1 blockade. A hallmark of the disease is the IgM kappa paraprotein, identified in 85% of the patients; recently variant IgG have been reported in 7% of cases.

## Objective

To characterise clinical symptoms in the 13 patients with SchS identified at the single UK centre. Recently, mosaicism in the *NLRP3* gene was described in two cases with SchS, which prompted us to search for mosaic variants in our cohort.

## Methods

13 patients underwent detailed clinical investigations and analysis of the *NLRP3* and *NLRP12* genes by Sanger and multiparallel sequencing (MPS). In addition *MYD88* gene was sequenced in the DNA extracted from whole blood.

## Results

The median age at disease onset was 55 years (range 35-78). All patients presented with uricarial rash, other manifestations included fever (77%), arthralgia (69%), weight loss (46%), fatigue (38%), bone pain (38%) and lymphadenopathy (23%). One patient was diagnosed with AA amyloidosis. In all subjects low grade IgM kappa paraprotein had been detected.

Genetic testing revealed two patients had V198M and F402L variants in the *NLRP3* and *NLRP12* genes respectively. No additional nucleotide alternations, including somatic mosaicism, in the *NLRP3* exons: 3, 4 and 6 have been identified by MPS. In addition no mutation was found in *MYD88* gene by PCR/Sanger sequencing.

## Conclusion

Despite of the recent report of *NLRP3* somatic mosaicism in two cases, in the current study, except for the two variants of unknown significance: V198M and F402L identified in the *NLRP3* and *NLRP12* genes respectively, no other genetic alternation was found by either Sanger or MPL sequencing in the 13 cases with SchS. We failed to identify variation in the *MYD88* gene, looking specifically for the L265P variant, which is a known risk factor for the development of Waldenstorm macroglobulinemia. The limitation of this study is that the analysis was performed on the DNA isolated from peripheral blood rather than the bone marrow (BM) and we plan to repeat this experiment on the BM samples.

